# Brassinosteroids in Plants: Crosstalk with Small-Molecule Compounds

**DOI:** 10.3390/biom11121800

**Published:** 2021-11-30

**Authors:** Dongliang Hu, Lijuan Wei, Weibiao Liao

**Affiliations:** College of Horticulture, Gansu Agricultural University, Lanzhou 730070, China; h1468009331@163.com (D.H.); wlj920229@163.com (L.W.)

**Keywords:** stress response, nitric oxide, ethylene, hydrogen peroxide, hydrogen sulfide

## Abstract

Brassinosteroids (BRs) are known as the sixth type of plant hormone participating in various physiological and biochemical activities and play an irreplaceable role in plants. Small-molecule compounds (SMCs) such as nitric oxide (NO), ethylene, hydrogen peroxide (H_2_O_2_), and hydrogen sulfide (H_2_S) are involved in plant growth and development as signaling messengers. Recently, the involvement of SMCs in BR-mediated growth and stress responses is gradually being discovered in plants, including seed germination, adventitious rooting, stem elongation, fruit ripening, and stress responses. The crosstalk between BRs and SMCs promotes plant development and alleviates stress damage by modulating the antioxidant system, photosynthetic capacity, and carbohydrate metabolism, as well as osmotic adjustment. In the present review, we try to explain the function of BRs and SMCs and their crosstalk in the growth, development, and stress resistance of plants.

## 1. Introduction

Indole-acetic acid (IAA) and gibberellin have been recognized as the known plant hormones found in plants many decades ago. Some studies have recently demonstrated that various phytohormones such as cytokinins (CTK), abscisic acid (ABA), ethylene, strigolactone, and melatonin are involved in plant growth and development and in responses to stress [1,2,3,4]. Brassinosteroids (BRs), a new type of plant hormone, have drawn an increased amount of attention. BRs, as a steroidal phytohormone, have been found to be involved in a wide range of physiological processes in plants, including cell elongation, cell division, seed development, flowering, and senescence, as well as both abiotic and biotic stress responses [5,6,7,8]. In addition, BRs have also been found to interact with other plant hormones to regulate plant growth and development as well as stress resistance. For example, co-treatment of melatonin and BRs significantly improved the resistance of *Festuca arundinacea* Schreb. to heat stress by decreasing the reactive oxygen species (ROS) level and malondialdehyde (MDA) content and increasing chlorophyll content and antioxidant enzyme activities [9]. In addition, studies involving BR-insensitive and BR-deficient mutants in the model plant *Arabidopsis thaliana* increasingly indicate that BRs might be vital endogenous growth modulators in plants. Meanwhile, BR loss-of-function mutants have also shown similar phenotypes, such as a dark-green color, obvious dwarfism, and a de-etiolation phenotype when grown in the dark [10]. She et al. elucidated the BR structure and found that kinase BRASSINOSTEROID INSENSITIVE 1 (BRI1) is the receptor of BRs [11]. They also further provided detailed molecular insights into BR recognition [11]. 

Different kinds of molecules play an essential role in transmitting information between cells of multicellular organisms, including small-molecule compounds (SMCs). The SMCs are produced and induced by signals in cells and then covalently bind to target cell receptors to cause multiple biological processes and stimulate responses both in animals and plants [12]. In the past, SMCs, such as nitric oxide (NO), hydrogen sulfide (H_2_S), and carbon monoxide (CO), were widely known for their toxicity. Their function in numerous plant growth and development processes is an inspiringly new development. Various studies have demonstrated the function of SMCs on a wide range of developmental and physiological processes, from root formation to postharvest senescence. Niu et al. suggested that NO promoted adventitious rooting in cucumber by protein post-translational modification (*S*-nitrosylation) [13]. Further, H_2_S at proper doses also improved the longevity and quality of cut roses and chrysanthemums by maintaining water balance, reducing the degradation of pigments and nutrients and enhancing antioxidant capacity [14]. As a class of abundant membrane components and signaling molecules, sphingosines increased the embryo biomass in *Gossypium hirsutum* Linn [15]. Additionally, SMCs have been proven to resist abiotic stresses in plants [16,17]. 

In recent years, an increasing number of SMC types have been indicated to interact with BRs. For example, BRs have been shown to interact with some typical small molecules such as NO, ethylene, hydrogen peroxide (H_2_O_2_), and H_2_S to modulate plant growth and tolerance to stress stimulus [18,19,20,21]. This provides further insight into the function mechanism of SMCs and a new type of plant hormones in plant growth and development processes. 

Some recent reviews have summarized the roles of steroidal phytohormone BRs in plants, which were mainly involved in the discovery of BRs and hormonal interactions in plant development and stress adaptation [8,22]. The roles of SMCs in adventitious rooting have also been reviewed [23]. However, the crosstalk between BRs and SMCs in plant growth and stress responses remains to be explored and reviewed. Therefore, for a better understanding of the functional mechanisms of BRs and SMCs in plants, we review recent works about the discovery and development of BRs and their interrelationship with SMCs in the growth, development, and stress responses of plants, which will provide directions for further work in this field. Finally, we discuss further perspectives to obtain a clear outlook of the crosstalk between BRs and SMCs.

## 2. Discovery and Development of Brassinosteroids

### 2.1. Discovery and Biosynthesis

Brassinosteroids (BRs), a class of essential steroidal phytohormones, are involved in many physiological and biochemical processes in plants. Brassinolide (BL), the most active BR, was first isolated from *Brassica napus* pollen in 1979, and the chemical structure of the substance was determined by crystal diffraction analysis [24]. With in-depth research in recent decades, BRs are generally considered to be the sixth most important plant endogenous hormone besides auxin, gibberellin, cytokinin, abscisic acid, and ethylene [25]. To date, about 70 naturally occurring compounds similar to BL have been isolated, and they are collectively referred to as BRs [26]. 

**Figure 1:**
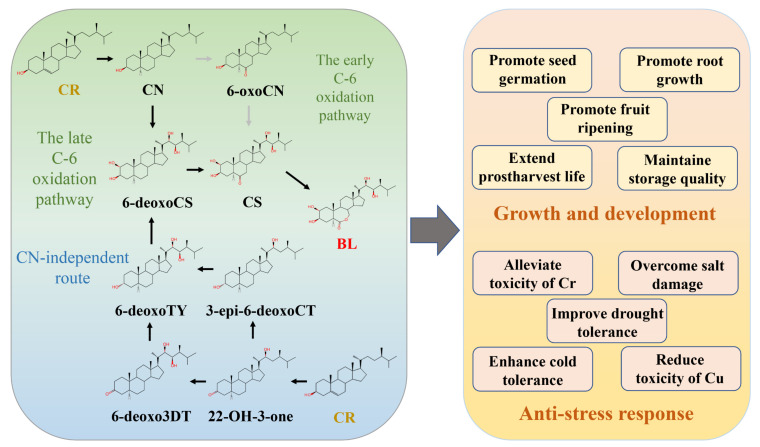
The biosynthetic pathway and roles of brassinosteroids (BRs) in the growth, development, and stress response of plants. The campestanol (CN)-dependent pathway: In the early C-6 oxidation pathway, CN is converted to 6-oxocampestanol (6-oxoCN), and 6-oxoCN is then converted to castasterone (CS). Additionally, in the late C-6 oxidation pathway, CN is converted to 6-deoxocastasterone (6-deoxoCS), which is converted to CS. The CN-independent pathway: 22-hydroxycampest-3-one (22-OH-3-one) is converted to 6-deoxo-3-dehydroteasterone (6-ddeoxo3DT) and 3-epi-6-deoxocastasterone (3-epi-6-deoxoCT) on different branches, and they are converted to 6-deoxotyphasterol (6-deoxoTY). The final synthesis product of different pathways is BL. BRs regulate plant growth and development by promoting seed germination, root growth, and fruit ripening, extending postharvest, and maintaining storage quality. Additionally, BRs are able to respond to different types of stress: alleviating the toxicity of Cr, overcoming salt damage, improving drought tolerance, enhancing cold tolerance, and reducing the toxicity of Cu.

The biosynthetic pathway of BRs is initiated by campesterol (CR). BR biosynthetic pathways are divided into a campestanol (CN)-dependent route (the early and the late C-6 oxidation pathways) and a CN-independent route (the early C-22 and C-23 hydroxylation pathways) [26]. In the early C-6 oxidation pathway, CN is converted to 6-oxocampestanol (6-oxoCN), and 6-oxoCN is then converted to CS. Additionally, in the late C-6 oxidation pathway, CN is converted to 6-deoxocastasterone (6-deoxoCS), which is converted to CS. In the CN-independent pathway, 22-hydroxycampest-3-one (22-OH-3-one) is converted to 6-deoxo-3-dehydroteasterone (6-ddeoxo3DT) and 3-*epi*-6-deoxocastasterone (3-*epi*-6-deoxoCT) on different branches, and they are then converted to 6-deoxotyphasterol (6-deoxoTY) [26]. The final synthesis product of different pathways is BL (Figure 1). A previous study indicated that the CN-independent and the late C-6 oxidation route made up the predominant biosynthetic pathway of BRs [27]. 

In *Arabidopsis thaliana*, BRs bound to BRI1, a receptor of BRs on the plasma membrane, and the activation of BRI1 then generated a phosphorylation cascade with its co-receptor BAK1 [8]. *BRASSINAZOLE RESISTANT 1* (*BZR1*) and *BRI1*-*EMS SUPPRESSOR 1* (*BES1*) have been recognized as two key transcription factors of BR signaling. *BRASSINOSTEROID*-*INSENSITIVE 2* (*BIN2*) is a negative modulator in the BR signaling pathway. The inactivated-*BIN2* made *BZR1* and *BES1* enter into the nucleus and regulated the expression of target genes in BR signal transduction, further positively regulating BR signaling [22]. In addition, a growing body of evidence indicates that both BAK1 and BRI1 play an indispensable role in BR signaling during plant growth and development. The overexpression of BAK1 led to an elongated organ phenotype in *Arabidopsis thaliana*, whereas a null allele of BAK1 presented semidwarf phenotypes and showed decreased sensitivity to BRs, further indicating that BAK1 is a significant component of BR signaling [10]. Wang et al. suggested that BAK1 and BRI1 interacted in vitro and in vivo, and the sequential transphosphorylation of BRI1/BAK1 affected early events in the BR signaling pathway [28].

### 2.2. The Roles of BR in Growth, Development, and Stress Response

BRs play a crucial role in plant growth and development processes such as seed germination, root development, fruit ripening, fruit fresh-keeping, and anti-aging [25,29]. The interaction of *BES1* with *ABSCISIC ACID INSENSITIVE5* (*ABI5*; an ABA transcription factor) significantly inhibited the combination of *ABI5* and the promoter regions of downstream genes, consequently suppressing ABA signaling output and promoting seed germination in *Arabidopsis thaliana* (Figure 1) [30]. In addition, BRs promoted root growth through *BZR1*-mediated transcriptional responses in *Arabidopsis thaliana*. Recently, Li et al. showed that BRs were dependent on *BIN2* and/or its downstream components *BZR1/BES1* to promote root development in Arabidopsis thaliana [25]. Thus, BRs regulate seed germination and root development through *BES1*-mediated transcription. Moreover, 10 µM (24-epibrassinolide) EBR promoted fruit ripening by enhancing ethylene biosynthesis and the activities of cell-wall-degrading enzymes in *Diospyros kaki* L. [31]. In *Solanum lycopersicum* L., the expression of *SlCYP90B3* was positively correlated with carotenoid accumulation and ethylene production [32]. Additionally, EBR treatment could maintain the membrane integrity of daylily flower buds and extend their postharvest life by delaying the degradation of chlorophyll, decreasing MDA content and electrolyte leakage [33]. EBR treatment (5 µM) delayed the senescence of kiwifruit and maintained their storage quality by increasing total soluble solid content and promoting the activity of superoxide dismutase, catalase, peroxidase, and ascorbate peroxidase (Figure 1) [29,34]. Thus, BRs might have different effects on the biosynthesis of fruit-ripening-related enzymes and pigments under different concentrations or in different plants. 

BRs are also involved in stress responses such as heavy metals, drought, salinity, high temperature, and low temperature [22]. Jan et al. indicated that the application of EBR alleviated the toxicity of chromium (Cr) in *Solanum lycopersicum* L. by modulating activities of antioxidant enzymes and ascorbate–glutathione cycle and by maintaining the glyoxalase cycle (Figure 1) [35]. Meanwhile, in *Zea mays* L. seedlings, EBR reduced MDA content and significantly increased osmoprotectants (proline, glycine betaine, and mannitol) to overcome the oxidative damage under salt stress [36]. *TaBZR2* (a BR transcription factor) interacted with the gene promoter to activate the expression of *TaGST1*, and the *TaGST1* could decrease superoxide anions (O_2_^−^) to contribute to drought tolerance in *Triticum aestivum* [37]. Moreover, EBR enhanced the cold tolerance of *Elymus nutans* by increasing proline content, decreasing MDA and ROS accumulation [38]. A previous study suggested that seed priming with EBR, nitrogen supplementation, and a combination of both could improve the activities of antioxidative enzymes to further decrease the lipid peroxidation and H_2_O_2_ generation under normal and salt stress in soybean [39]. In summary, BRs could resist different abiotic stresses through modulating the antioxidative system. Nazir et al. showed that EBR and H_2_O_2_ ameliorated the chloroplast ultrastructure and stomatal behavior to improve photosynthetic efficiency, thus decreasing the toxicity of copper (Cu) in *Solanum lycopersicum* [40]. EBR could alleviate the negative effects of salt stress on *Solanum tuberosum* L. by improving the content of photosynthetic pigments, photosynthetic electron transport, the photosystem II (PSII) maximum, and effective quantum yields [41]. Similar results were reported by Junior et al. who found that EBR treatment increased the photosynthetic rate, the transpiration rate, and stomatal conductance to involve in drought stress response in *Eucalyptus urophylla* (Figure 1) [42]. Additionally, the application of EBR enhanced the chilling stress tolerance of *Piper nigrum* L. through maintaining the photosynthetic rate, the maximum quantum efficiency (Fv/Fm), and the photochemical quenching coefficient [43]. Therefore, BRs might improve plant abiotic stress tolerance by regulating the photosynthesis mechanism. 

A recent study found that the application of EBR and salicylic acid (SA), as well as silicon (Si), significantly decreased the content of H_2_O_2_, MDA, and EL to improve the growth and quality of *Triticum aestivum* L. under arsenic (As) stress [44]. BIP130 (a BRI1-interacting protein) enhanced the salt stress tolerance in *Oryza sativa* L. through regulating abscisic acid (ABA) biosynthesis and scavenging ROS [45]. An exogenous application of EBR increased the content of BRs and decreased the level of ABA and ROS, after which drought resistance in *Solanum lycopersicum* improved [46]. Interestingly, Choudhary et al. indicated that BR signaling increased NO levels, which in turn triggered ABA biosynthesis and promoted the growth of *Raphanus sativus* seedlings [47]. These studies imply that BRs might improve abiotic stress tolerance via interacting with other phytohormones and/or be involved in the biosynthetic pathway of other phytohormones, providing better evidence of the relationships of BRs and other phytohormones when plants are subjected to abiotic stress.

In addition, BRs play a crucial role in lignin accumulation, which decreased the toxicity of salt stress in *Allium sativum* L. [48]. The interaction of EBR and Si could improve the high-temperature tolerance of *Triticum aestivum* L. by elevating the antioxidant system and osmoprotectant [49]. Li et al. showed that exogenous EBR treatment increased the accumulation of theanine in *Camellia sinensis* L. to improve the quality of summer tea under high-temperature conditions [50]. These findings expand the understanding of the response mechanism under abiotic stress in plants. 

Moreover, BRs have also been found to participate in the biotic stress response. For example, BL enhanced the content of NO and further decreased the accumulation of *cucumber mosaic virus* (CMV) in *Arabidopsis thaliana* [51]. In Gossypium spp., the application of EBR could alleviate *Verticillium dahlia* (Vd) toxins mostly by improving the content of photosynthetic pigments and regulating secondary metabolism [52]. Therefore, the BR-mediated biotic stress response might be related to the photosynthetic mechanism and secondary metabolism. In addition, BRs might play a crucial role in stress response through a complex series of biochemical reactions. In the future, BRs will come to be known as an irreplaceable phytohormone. 

In general, the studies mentioned above indicate the vital role of BRs in the growth, development, and stress response of plants. That is to say, BRs could respond to stress stimulus and promote growth and development in various ways, such as the regulation of the antioxidant system and the photosynthesis mechanism as well as interactions with plant hormones in plants. Research on this subject contributes to diversely and effectively understanding the roles of BRs in growth, development, and stress response in plants. In addition to interacting with phytohormones, BRs have also been found to interact with SMCs in plants, including NO, ethylene, H_2_O_2_, and H_2_S. Thus, in the following sections of this review article, we mainly focus on the current state of knowledge regarding the interaction between BRs with SMCs in plants.

## 3. Brassinosteroids and Nitric Oxide

**Figure 2:**
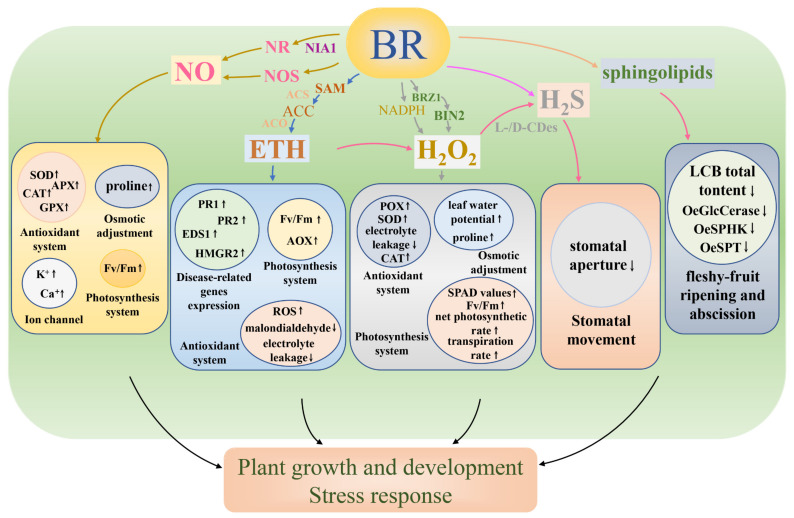
Model of the signal pathways by which brassinosteroids (BRs) crosstalk with other SMCs in the growth, development, and stress response of plants. In terms of interaction with nitric oxide (NO), BRs induce NO generation through nitrate reductase (NR) and NO synthase (NOS) pathways. As the main synthesis pathway, NITRATE REDUCTASE1 (NIA1) is responsible for BR-induced NO through the NR pathway. The crosstalk between BRs and NO regulates the antioxidant system, the photosynthesis system, osmotic adjustment, and the ion channel. Additionally, BRs are involved in the biosynthesis of ethylene through S-adenosyl-L-methionine (SAM) and 1-aminocyclopropane-1-carboxylic acid (ACC) and enhance the activity of key enzymes ACC oxidases (ACO) and ACC synthases (ACS) in the synthesis pathway to promote ethylene production. The crosstalk between BRs and ethylene is involved in the antioxidant system and the photosynthesis system and upregulates the expression of disease-related genes. In terms of hydrogen peroxide (H_2_O_2_), BR signaling through BRASSINOSTEROID INSENSITIVE 1 (BRI1) triggers the production of H_2_O_2_ in the NADPH-dependent pathway, and H_2_O_2_ regulates the BR activity downstream of BRASSINOSTEROID-INSENSITIVE 2 (BIN2). The crosstalk between BRs and H_2_O_2_ regulates the antioxidant system, the photosynthesis system, and osmotic adjustment. Furthermore, ethylene modulates BR-mediated stomatal closure via inducing H_2_O_2_. Hydrogen sulfide (H_2_S) further functions as the downstream of H_2_O_2_. Meanwhile, BRs downregulate the sphingolipid long-chain base (LCB) total content and the expression levels of sphingolipid-related genes {the serine palmitoyltransferase I (OeSPT); sphingosine kinase (OeSPHK); glucosylceramidase (OeGlcCerase)}.

NO, a redox-related small gas molecule, has indispensable effects on various biological systems. Generally, NO production is mainly through nitrate reductase (NR) and NO synthase (NOS) pathways (Figure 2) [16]. More recently, increasing studies indicate that NO is involved in multiple growth and development processes, including seed germination, senescence of cut roses, adventitious root development, and stomatal closure [53,54,55,56]. Simultaneously, NO plays an essential role in plant responses to multiple abiotic stresses [57]. The involvement of NO in osmotic stress, heavy metal stress, drought stress, heat stress, chilling stress, and salt stress has been elucidated [16,58,59,60,61,62]. Moreover, under water-deficit stress, sodium nitroprusside (SNP, a NO donor) decreased the incidence of *tobacco mosaic virus* (*TMV*) and *tomato yellow leaf curl virus* (*TYLCV*) in *Solanum lycopersicum* [63]. Thus, as a small gas molecule, its roles in plant growth and stress response might be a great topic of interest. More importantly, recent studies found that BRs might interact with NO, which plays an essential role in plant growth and stress response (Table 1 and Table 2). Recently, Karpets et al. found that the co-treatment with EBR and SNP in low concentrations significantly enhanced the heat resistance of *Triticum aestivum* L. [64]. However, these authors also mentioned that this synergistic effect might be only in the relatively narrow range of concentrations of NO and BR donors, and high concentrations might reduce their protective roles under stresses. In addition, BRs enhanced tolerance to salt stress, whereas the NO scavenger, 2-(4-carboxyphenyl)-4,4,5,5-tetramethyl-imidazoline-1-1-oxyl-3-oxide (cPTIO) applications, or the virus-induced gene silencing of NR and NOS-like enzymes inhibited BL-induced salt resistance in *Nicotiana benthamiana* seedlings [65], revealing that NO may play essential roles in BR-induced salt tolerance. The root system plays a significant role in the transmission of the signal to branches and leaves [66]. Li et al. found that BL promoted the formation of adventitious roots by inducing the production of endogenous NO in *Cucumis sativus* L. [67]. Further, EBR increased the NO levels in root cells, which in turn NO was essential for root architecture in *Arabidopsis thaliana* [68]. The burst of NO triggered by EBR might have a positive effect on the root development and growth in *Arabidopsis thaliana*, which might be because this EBR-induced NO burst affected stomatal closure [69]. Intriguingly, NO was required for the EBR-triggered increase of flavonoid biosynthesis in tea leaves, which further improved the quality of green tea [70]. Using pharmacological and biochemical approaches, Ren et al. unveiled that NO and BL promoted the fungal endophyte-induced production of volatile oil through protein phosphorylation in *Atractylodes lancea* plantlets, therefore activating secondary metabolites and improving the medicinal value of *Atractylodes lancea* [71]. This implies that the interaction of BRs and NO plays a vital role in regulating the quality of plants, which might be a potential and important line of inquiry in improving crop quality in the future.

Additionally, a growing body of evidence pointed out that BRs could regulate endogenous NO levels in different ways to affect plant growth and stress resistance (Table 1 and Table 2). According to pharmacological and genetical evidence, Tossi et al. revealed that EBR treatment increased NO production by inducing NR and NOS-like in *Arabidopsis thaliana* [66], which further enhanced lateral root density. These effects were verified by adding NO donor S-nitrosoglutathione (GSNO) to BRI1-1, a BR receptor mutant [66]. Interestingly, Kaya et al. found that EBR enhanced the tolerance to iron deficiency in *Fragaria* × *ananassa* by increasing leaf Fe^2+^ content and the activities of antioxidant enzymes, leading to a further increase in the NO level and in NR and NOS-like activity [72]. Thus, NR, rather than NOS, participated in BR-induced NO production and enhanced iron deficiency tolerance in *Fragaria* × *ananassa*. Similarly, EBR could induce NO generation via the NR pathway in *Capsicum annuum* L., which further alleviated Cd stress by promoting the antioxidant enzymes and the ASA-GSH cycle [73]. In *Arabidopsis thaliana*, NR was encoded by *NITRATE REDUCTASE1* (*NIA1*) [81]. BL promoted NO accumulation and reduced virus accumulation in *Arabidopsis thaliana* but did not increase NO content in *nia1* mutants [51]. They also found that, compared with wild-type plants, *nia1* mutants exhibited decreased virus resistance after BL treatment, indicating that NR-dependent NO production was responsible for BR-mediated virus resistance in *Arabidopsis thaliana*. Consequently, these studies revealed that BRs could activate NR and/or NOS to trigger endogenous NO, which could act as a downstream signal molecule in the growth and development of plant stress response. Furthermore, in most cases, the NR pathway might be the main pathway in BR-induced NO biosynthesis (Figure 2).

Together, the interaction between BRs and the small gas molecule NO has an essential role in the growth, development, and stress response of plants. However, the specific mechanism of their interaction is still not clear and needs further study. Further, *S*-nitrosylation, a redox-based posttranslational modification, is an NO-dependent regulatory mechanism. Thus, whether BRs interact with NO through protein *S*-nitrosylation in the BR signaling pathway might warrant further attention.

## 4. Brassinosteroids and Ethylene

Ethylene is a simple gaseous plant hormone that consists of two carbon and four hydrogen atoms. It is synthesized in almost all plant tissues and organs. It affects key physiological processes and stress responses in plants. Ethylene biosynthesis begins with methionine and forms the end product through three main steps. 

The “triple response” of ethylene on etiolated seedlings is well known. Similarly, Jiroutová et al. also found that BRs inhibited the growth of *Pisum sativum* L. seedings, along with a reduction in stem elongation rate, an increase in lateral expansion, and an exaggeration of the apical hook curvature. Subsequently, they demonstrated that BRs promoted the biosynthesis of endogenous ethylene, and the inhibitory effect of BRs was mediated by ethylene [19]. In addition, it was found that, compared with wild-type fruit, a higher ethylene content was obtained in the *SlCYP90B3*-OE fruit [the transgenic lines overexpressing *SlCYP90B3* (a BR transcription factor) of *Solanum lycopersicum*]. A further study found that the expression level of ethylene biosynthetic genes (*SlACS2*, *SlACS4*, and *SlACO1*) and signaling genes (*SIETR3* and *SICTR1*) was significantly upregulated in *SICYP90B3*-OE transgenic lines [32]. These studies indicated that *SICYP90B3*-OE enhanced ethylene production in *Solanum lycopersicum* fruit. Jiang et al. suggested *BES1*, a BR transcriptional factor, controlled the level of endogenous ethylene in *Arabidopsis thaliana* by regulating the expression of ACO2 [1-aminocyclopropane-1-carboxylic acid (ACC, the direct precursor of ET) oxidase 2] [78]. Taken together, BRs may participate in the ethylene biosynthetic pathway by regulating ethylene biosynthetic genes and signaling genes as well as ethylene biosynthesis-related enzymes (Figure 2 and Table 2). 

Under abiotic stress conditions, the alternative oxidase (AOX) could eliminate the superfluous accumulation of BL-mediated ROS to protect photosystems and thus enhanced the stress tolerance of *Nicotiana benthamiana* [82]. Wei et al. reported that BL increased the production of ethylene and the expression level of AOX in *Cucumis sativus* L. seedlings under drought, salt, and chilling stresses [74]. Pretreatment with the ethylene biosynthesis inhibitor aminooxy acetic acid (AOA) significantly decreased the BL-induced resistance of photo-oxidation in seedlings, whereas the negative roles could be reversed by ethylene [74]. Thus, the interaction between BRs and ethylene alleviates the oxidative damage in the plant photosystem to enhance tolerance under stress conditions. Meanwhile, ethylene also has a positive effect on the BR-enhanced resistance of abiotic stresses in plants.

It is well known that both BRs and ethylene play a positive role in biotic stresses (Table 1). In *Nicotiana benthamiana*, the treatment of BL and ACC increased the resistance to *Pseudomonas syringae* pathovar tomato DC3000 (*Pst* DC3000) and inhibited the growth of pathogenic bacteria. Meanwhile, ACC treatment significantly increased the content of callose deposition, improved the activities of antioxidant enzymes and ROS accumulation, and activated the expression of four disease-related genes (*PR1*, *PR2*, *EDS1*, and *HMGR2*) [75]. Sequentially, they found that the silence of the BR biosynthetic gene *DWF4*, the BR receptor BRI1, the downstream gene of BRI1 (*BSK1*), and the application of BRZ (a specific BR biosynthetic inhibitor) all led to the counteraction of ethylene-induced resistance. Interestingly, they found that aminoethoxyvinylglycine (AVG), an ethylene biosynthetic inhibitor, inhibited ethylene biosynthesis, while there was no effect on BR-induced resistance [75]. Therefore, BRs might be involved in ethylene-induced biotic stress resistance by enhancing callose deposition, ROS accumulation, and the activities of antioxidant enzymes as well as the expression of disease-related genes in a BR-dependent way (Figure 2).

Overall, BRs can participate in ethylene biosynthetic genes, signal transduction, and related enzymes. Ethylene can be involved in the growth, development, and stress responses in a BR-dependent way. Given the importance of ethylene for the postharvest of crop products, the interactions between BRs and ethylene have great prospects for the future.

## 5. Brassinosteroids and Hydrogen Peroxide

H_2_O_2_, a crucial small signaling molecule, affects the physiologic and biochemical processes in plants. As an ROS, H_2_O_2_ is generated at the cell surface, which may regulate plant growth and stress response at low concentrations. Salama et al. showed that the application of 600 ppm H_2_O_2_ increased growth and yield in *Solanum tuberosum* by enhancing root respiration and the content of chlorophyll and soluble carbohydrates under drought stress [83]. At elevated levels, H_2_O_2_ can cause oxidative burst to destroy the structure of some proteins and further interfere with the signal transmission process of cells [40]. In recent years, studies on the crosstalk between H_2_O_2_ and BRs have become more popular.

Both H_2_O_2_ and BRs participate in plant developmental processes as signaling messengers, so it is important to know how the crosstalk between H_2_O_2_ and BRs functions in plants (Table 2). A previous study suggested that H_2_O_2_ regulated photosynthesis in an EBR-mediated way, and the crosstalk between EBR and H_2_O_2_ was involved in sugar metabolism and the Calvin cycle in *Cucumis sativus* through a redox signaling pathway [79]. Tian et al. showed that H_2_O_2_ content was significantly improved in BR-treated *Arabidopsis thaliana* seedlings, and a BR-induced H_2_O_2_ level was triggered through an NADPH-dependent pathway (Figure 2) [20]. Thereafter, they evaluated whether H_2_O_2_ played a potential role in BR-mediated seedling development. They showed that diphenylene iodonium (DPI, the inhibitor of NADPH oxidase) treatment decreased H_2_O_2_ levels and significantly inhibited the effects of BRs on hypocotyl elongation. Meanwhile, high concentrations of DPI led to an insensitivity to BRs in *Arabidopsis thaliana* seedlings [20]. In general, BRs and H_2_O_2_ might enhance crop yield by regulating photosynthesis and sugar metabolism. In addition, BR might improve endogenous H_2_O_2_ levels in plants, further enhancing BR-mediated plant cell elongation.

BR and H_2_O_2_ co-treatment could improve plant resistance to abiotic stresses (Table 1). In *Lycopersicon esculentum*, the application of EBR and H_2_O_2_ significantly increased SPAD chlorophyll, the net photosynthetic rate, and the activity of carbonic anhydrase and different antioxidant enzymes (CAT and SOD) under cold stress [76]. Heavy metals at high concentrations are harmful to plant tissues and organs. Nazir et al. investigated whether the combination of BRs and H_2_O_2_ can reduce the toxicity of Cu in *Solanum lycopersicum* [40]. They found that the co-treatment of EBR and H_2_O_2_ had significantly increased chlorophyll content and Fv/Fm compared with EBR or H_2_O_2_ alone. EBR and H_2_O_2_ increased the net photosynthetic rate and related traits (the internal carbon dioxide concentration, stomatal conductance, and the transpiration rate) and reduced the electrolyte leakage. Cu treatment decreased the leaf area and dry mass of shoots and roots in tomato seedlings, while the combined application of EBR and H_2_O_2_ significantly increased these parameters. Similarly, EBR and H_2_O_2_ also modified the chloroplast ultrastructure and stomatal behavior and increased the total protein content and the activities of antioxidant enzymes and carbonic anhydrase in Cu-treated tomato seedlings under Cu stress [40]. Thus, the interaction between BRs and H_2_O_2_ might enhance photosynthetic capacity and total protein content and might maintain the antioxidant system and plasma membrane, thereby increasing plant resistance to abiotic stress. In *Nicotiana benthamiana*, Deng et al. indicated that BRs increased the resistance of TMV [77]. However, pretreatment with dimethylthiourea (DMTU), a scavenger of H_2_O_2_, decreased the tolerance of TMV in *Nicotiana benthamiana* (Figure 1). Therefore, BR-mediated virus resistance requires H_2_O_2_, which participates in the regulation of virus resistance. Overall, H_2_O_2_ plays an important role in BR-induced growth, development, and stress responses. Additionally, H_2_O_2_ might regulate the complex signaling network mechanism as a downstream signaling messenger in BR signaling in the growth and stress responses of plants. However, many theoretical mechanisms of the interaction between BRs and H_2_O_2_ are still unclear, so further research and discoveries are needed.

## 6. Brassinosteroids and Hydrogen Sulfide

H_2_S is an endogenous biological signal molecule with a unique odor of rotten eggs. H_2_S is known to be a poisonous gas, and its toxicity has always been a focus of research. In recent years, research on H_2_S has been increasingly concerned with its roles in plant growth, development, and stress response [84,85]. As a second signaling messenger, the interaction between H_2_S and BRs might play a crucial role in plants.

In *Arabidopsis thaliana*, the application of methyl jasmonate (MeJA) decreased stomatal density in wild-type seedlings. However, the treatment of hypotaurine (HT, a scavenger of H_2_S) could eliminate the negative roles of MeJA-reduced stomatal density in the wild type [86]. In addition, a previous study suggested that H_2_S, downstream of phytohormone salicylic acid (SA), enhanced the chilling tolerance in *Cucumis sativus* L. seedlings through regulating the antioxidant system [87]. These findings suggest that the phytohormone regulated stomatal development and improved cold tolerance through an H_2_S-dependent pathway. Ma et al. found that EBR treatment alone led to the stomatal closure in *Arabidopsis thaliana*. Subsequently, they found that HT, AOA, and hydroxylamine (NH_2_OH) (the H_2_S biosynthesis inhibitors) as well as C_3_H_3_KO_3_ + NH_3_ [the producer of L-/D-cysteine desulfhydrase (L-/D-CDes)] could significantly inhibit EBR-mediated stomatal closure [21]. Moreover, the application of EBR significantly improved L-/D-CDes activity (the major enzymes that catalyze the degradation of cysteine into H_2_S) and H_2_S content. However, HT, AOA, NH_2_OH, and C_3_H_3_KO_3_ + NH_3_ could lessen the EBR-induced increase of the activity of L-/D-CDes and the content of H_2_S (Figure 1). Thus, H_2_S might be involved in EBR-induced stomatal closure (Table 2) [21]. Thus, H_2_S might play an irreplaceable role in BR-mediated stomatal movement and the photosynthetic system. Overall, H_2_S, as a signaling molecule downstream of the BR signaling transduction pathway, participates in plant growth and development, and H_2_S as a downstream signal molecule in other plant hormones may enhance abiotic stress tolerance, which may be important to provide new insights into how the combined effect of H_2_S and BRs is involved in abiotic and biotic stress responses in plants.

## 7. Brassinosteroids and Sphingolipids

Sphingolipids are an essential component of plant biomembranes. Sphingolipids have been extensively studied in animals and yeast and have been proved to be a class of active molecules. Sphingolipids are involved in cell growth, differentiation, senescence, and programmed cell death [88,89]. The roles of sphingolipids in plants have been studied in recent years.

Corbacho et al. observed the interaction between sphingolipids and BRs during the early fleshy-fruit growth in *Olea europaea* L. The application of exogenous EBR significantly reduced the total content of sphingolipid long-chain base (LCB) and the transcript levels of sphingolipid-related genes {the serine palmitoyltransferase I (*OeSPT*); sphingosine kinase (*OeSPHK*); glucosylceramidase (*OeGlcCerase*)}. However, BRZ application improved the sphingolipid LCB content and the gene expression [80]. Thus, BRs might negatively regulate the content of sphingolipids during fruit development. Sphingolipids could inhibit fruit growth, while BRs can alleviate the negative effects of sphingolipids. The crosstalk between BRs and sphingolipids might be extremely complicated. There is a need to conduct in-depth studies on the role of the interaction between BRs and sphingolipids in various crops.

## 8. Conclusions and Future Perspectives

As a natural plant hormone, BRs and its analogs, as the sixth class of phytohormones, are widely present in plant tissues and organs such as pollen, seeds, stems, and leaves. Since BRs were discovered, their regulatory mechanism and network in the growth, development, and environmental stress responses of plants have become increasingly clear. Meanwhile, NO, ethylene, H_2_O_2_, and H_2_S, as second signal messengers, also participate in plant growth and stress response. The interaction of BR signaling pathways with NO, ethylene, H_2_O_2_, and H_2_S makes up a complex regulatory network involving developmental processes, such as seed germination, root development, stomatal closure, and stem elongation. Their crosstalk may also enhance the plant tolerance of abiotic and biotic stresses such as heavy metals, drought, salt, heat, cold, pest, and disease. Furthermore, they positively co-regulate plant physiological and biochemical activities such as the antioxidant system, the photosynthesis system, the integrity of the plasma membrane, and the expression level of related genes. Emerging evidence suggests that environmental stresses and phytopathogens can induce hormone signaling transduction and plant defense mechanisms to prevent injury. However, we have limited knowledge on how the combined effect of BRs and various signaling molecules operate in complex regulatory mechanisms in plant growth and stress responses. Do they participate in maintaining the ion balance of the plasma membrane system? Are they involved in coding-related proteins? How do they repair damaged cells? Therefore, researchers need to explore the role of crosstalk between BRs and other small molecules in organisms.

## Figures and Tables

**Table 1:** The roles of BRs and small-molecule compounds under abiotic/biotic stresses.

Small-Molecule Compound	Type of BRs	Type of Stress	Plant Species	Plant Tissue	Effect	Reference
NO	EBR	Heat	*Triticum aestivum* L.	Seedlings	Improves the antioxidant system’s ability to enhance tolerance	[64]
	EBR	Iron deficiency	*Fragaria × annassa* Duch.	Leaves	Improves the antioxidant system’s ability to enhance tolerance	[72]
	BL	Salt	*Nicotiana benthamiana* L.	Seedlings	Enhances tolerance by playing a role in the photosystem	[65]
	EBR	Cd	*Capsicum annuum* L.	Leaves	Improves the antioxidant system’s ability and the ASA-GSH cycle	[73]
Ethylene	BL	Drought, salt, cold	*Cucumis sativus* L.	Seedlings	Increases the AOX activity to enhance photo-oxidative resistance	[74]
	BL	*Pst* DC3000	*Nicotiana benthamiana* L.	leaves	Improves the antioxidant system’s ability and activates the expression of disease-related genes	[75]
H_2_O_2_	EBR	Cold	*Lycopersicon esculentum*	Seedlings	Enhances the antioxidant system’s ability and the photosynthetic system	[76]
	EBR	Cu	*Solanum lycopersicum*	Seedlings	Enhances the antioxidant system’s ability and the photosynthetic system as well as the total protein content	[40]
	BL	TMV	*Nicotiana benthamiana* L.	Leaves	Enhances the systemic virus resistance	[77]

Note: “EBR”, “24-epibrassinolide”; “BL”, “Brassinolide”.

**Table 2:** The roles of BRs and small-molecule compounds in plant growth and development.

Small-Molecule Compound	Type of BRs	Plant Species	Plant Tissue	Effect	Reference
NO	BL	*Cucumis sativus* L.	Roots	BL-induced NO generation promotes adventitious root formation	[67]
	EBR	*Arabidopsis thaliana* L.	Roots	NO participates in EBR-induced changes in root architecture	[68]
	EBR	*Arabidopsis thaliana* L.	Roots	EBR-induced NO affects the stomatal closure of the root system	[69]
	BL	*Atractylodes lancea*	Plantlets	BL and NO activate secondary metabolites and improve the medicinal value	[71]
Ethylene	EBR	*Pisum sativum* L.	Seedlings	EBR-induced ethylene inhibits seedling growth	[19]
	-	*Solanum lycopersicum*	Fruits	The BR biosynthetic gene *SICYP90B3*-*OE* enhances ethylene generation to promote fruit ripening	[32]
	BL	*Arabidopsis thaliana* L.	Seedlings	The BR transcriptional factor *BES1* regulates the expression of ACO2 to maintain the level of endogenous ethylene	[78]
H_2_O_2_	EBR	*Cucumis sativus* L.	Seedlings	EBR and H_2_O_2_ co-regulate the sugar metabolism and Calvin cycle through the redox signaling pathway	[79]
	BL	*Arabidopsis thaliana* L.	Seedlings	BL-induced H_2_O_2_ promotes hypocotyl elongation	[20]
H_2_S	EBR	*Arabidopsis thaliana* L.	Leaves	H_2_S participates in EBR-induced stomatal closure	[21]
Sphingolipids	EBR	*Olea europaea* L.	Fruits	BRs negatively regulate sphingolipid content in fruit	[80]

Note: “EBR”, “24- epibrassinolide”; “BL”, “Brassinolide”; “-”, “No external treatment”.
